# Size Matters: A Large-Scale, Real-World Dataset of Smartphone-Based Obstructive Sleep Apnea Screening in the General Population

**DOI:** 10.3390/diagnostics16203364

**Published:** 2026-10-16

**Authors:** Christoph Janott, Marcel Braun, Torsten Eggert, Matthias Jakobs, Simone Salzger, Jörg Ulrich Sommer, Clemens Heiser, Christoph Schöbel

**Affiliations:** 1 Ruhrlandklinik, University Medicine Essen, 45239 Essen, Germany; 2 Klinikum Rechts der Isar, Technical University Munich, 81675 Munich, Germany; 3 HNO-Zentrum Mangfall-Inn, 83043 Bad Aibling, Germany

**Keywords:** obstructive sleep apnea, smartphone, screening, telemedicine, digital health, real-world data, longitudinal data, apnea–hypopnea index

## Abstract

**Background**: Obstructive sleep apnea (OSA) is a highly prevalent sleep-related breathing disorder that remains substantially underdiagnosed and is causally linked to cardiovascular and metabolic morbidity. Clinical diagnosis relies on the apnea–hypopnea index (AHI) derived from polysomnography (PSG) or home sleep apnea testing. Low-threshold, clinically validated screening tools that can be used repeatedly outside a clinical setting may help to reduce underdiagnosis and to capture longitudinal information. **Methods**: We describe a real-world dataset generated by a medical device-grade, smartphone-only OSA screening application (Snorefox M) that calculates the AHI from nocturnal breathing sounds. All users who provided informed consent for the research use of their anonymized data between March 2023 and December 2025 were included. The diagnostic performance of the application had previously been validated against parallel PSG. Additional post hoc analysis of classification performance at additional AHI thresholds, together with the linear correlation between the app-derived and PSG-derived AHI are described. **Results**: The dataset comprises 239,132 measurements from 31,647 consenting adults. A total of 14,351 users contributed at least two valid nights, yielding 221,820 nights of longitudinal screening data. Across all attempted measurements, 96.5% yielded a valid analysis result. The mean per-measurement AHI was 14.3 (median 11.8, SD 11.0). Voluntary, self-reported demographic information was available for a subgroup of 8092 users (mean age 54.4 years, mean ESS score 7.2). Against PSG, the application detected moderate-to-severe OSA (AHI ≥ 15) with a sensitivity of 0.91 and a specificity of 0.83, and severe OSA (AHI ≥ 30) with a sensitivity of 0.93 and a specificity of 0.97. The app-derived AHI correlated strongly with the PSG-derived AHI (Pearson r = 0.80, *p* < 0.001). The app-derived AHI discriminated against the PSG reference with an area under the curve (AUC) of 0.951 for severe OSA (AHI ≥ 30), 0.925 for moderate-to-severe OSA (AHI ≥ 15) and 0.877 for the AHI ≥ 10 threshold. **Conclusions**: This is, to our knowledge, the largest longitudinal dataset of unsupervised, real-world OSA screening from a general adult population using a clinically validated, low-threshold smartphone tool. Smartphone-based screening can complement established diagnostic pathways, support pre-clinical triage and generate longitudinal data not accessible through single-night testing, while self-selection and the unsupervised nature of data capture constrain representativeness. By introducing this resource, this work provides the basis for the in-depth analyses of demographics, symptom–result concordance and night-to-night variability.

## 1. Introduction

Obstructive sleep apnea (OSA) is among the most common chronic sleep-related breathing disorders, with recent global estimates placing the number of affected adults at close to one billion and identifying it as an important contributor to cardiovascular, metabolic and neurocognitive morbidity [1,2]. Despite this burden, the majority of affected individuals remain undiagnosed [2,3].

The contribution of OSA to cardiovascular disease is well established [4]. Its role in the development of systemic hypertension is particularly well documented [5], with OSA being present in close to 80% of patients with treatment-resistant hypertension [6,7]. Importantly, treating OSA with positive airway pressure lowers blood pressure, particularly in patients with resistant hypertension [6,7,8].

Beyond its cardiovascular consequences, OSA carries a considerable global health and economic burden and meaningfully reduces quality of life [9]. Simulation-based health-economic modeling of early OSA screening in a German population suggests favorable effects on quality of life, survival and overall healthcare costs [10].

Population-based data demonstrate that a substantial share of adults, on the order of one in five, harbor disease severe enough to warrant treatment, a figure that tracks the rising prevalence of obesity and the aging of the population [11].

Underdiagnosis compounds these challenges. Because OSA frequently presents with symptoms that those affected often do not associate with OSA, an estimated four out of five adults with moderate or severe disease remain undiagnosed, generating avoidable healthcare and societal costs [3,12]. Where the underlying disorder goes unrecognized, its consequences may instead be treated symptomatically over long periods, as in drug-resistant hypertension [6,7].

Several factors sustain this diagnostic gap: limited awareness of OSA in the general public [13] and in primary care [14], the burdensome nature of conventional diagnostic procedures, and the restricted capacity of the healthcare system, especially sleep laboratories, with correspondingly long waiting times [15].

The resulting demand for affordable, accessible methods of early detection has driven the development of smartphone-based screening solutions intended to broaden diagnostic reach and improve detection rates by leveraging hardware that is already in widespread use [16].

Because smartphones are already available to a large portion of the adult population, smartphone-based OSA screening aims to lower the barriers to assessment and to enable timely referral where indicated, with potential benefits for patient outcomes, quality of life and the efficient use of constrained healthcare resources [3].

Recent years have seen a proliferation of “wearable” and “nearable” solutions that allow the general population to self-monitor a range of sleep-related metrics, offering broad access to wellness tracking. The majority of these products, however, are consumer-grade and have not been validated against clinical reference standards, leaving their actual benefit for health-related decisions uncertain.

Because these data are often generated and viewed by medical laypersons, their wide availability carries both benefits and risks. Creating awareness in the broader population about the importance of sleep quality for general health and conveying information about possible causes of symptoms can generally activate those affected to take necessary action. On the other hand, false positive findings or the mere suggestion of a possible disorder may cause unnecessary alarm and prompt avoidable physician visits by worried users, potentially putting an additional load on the healthcare system [17]. Data that are misinterpreted as reassuring may lull users into a false sense of security, so that an existing but occult condition goes unnoticed.

Conversely, population-wide screening with low-threshold, widely available tools that have been validated against clinical diagnostic standards can help to reduce the number of undetected cases and to enable more effective triage of patients before their first contact with a physician, or within primary care prior to specialist referral, thereby supporting more efficient use of limited healthcare resources.

An additional value can lie in the aggregated, anonymized data collected by means of such tools, which can contribute valuable population-based information on prevalence, the relationship of sleep disorders and sleep behavior, and even longitudinal information about disease development over longer periods of time.

This paper pursues three objectives. First, it describes the methodology and feasibility of touchless, smartphone-based OSA screening using a medically validated smartphone application for low-threshold, broadly accessible OSA screening and the manner in which the data are collected. Second, it summarizes and extends the validation of the underlying measurement against the clinical gold standard. Third, it introduces the resulting dataset from nearly three years of population-level data collection in Germany, characterizes it in terms of real-world data quality, and critically appraises its validity, value and limitations.

To our knowledge, this is the first description of a large-scale, real-world dataset on OSA-related information drawn from the German general population. The present work is therefore conceived as a resource paper: it introduces the dataset, describes its properties and demonstrates its validity as a necessary precondition for further analysis built upon it. In-depth investigations of user demographics, the relationship between self-reported symptoms and screening results, and longitudinal, multi-night use are the subject of dedicated analyses that are in preparation by our group and are beyond the scope of this paper.

## 2. Materials and Methods

### 2.1. The Snorefox M Screening Application

Snorefox M (Version 1, Diametos GmbH, Potsdam, Germany) is a software-only smartphone application for screening the risk of OSA. It runs on a range of Apple and Android handsets that have passed the manufacturer’s compatibility testing. During the night, the application uses the smartphone’s built-in microphone to record and characterize the patterns of nocturnal breathing sounds. From these acoustic patterns, the Snorefox M algorithm identifies respiratory anomalies and derives an estimate of the AHI for the recorded night.

The application is a CE-marked medical device under the European Medical Device Directive (MDD). It is intended for use by adult medical laypersons to assess the risk of OSA as part of a preliminary examination, i.e., screening, and is explicitly not intended to provide a diagnosis; the definitive diagnosis or exclusion of a sleep-related breathing disorder remains the responsibility of the attending physician within a guideline-based work-up.

In Germany, the application is available to lay users through the Apple App Store and the Google Play Store and does not require a medical prescription, which makes it widely accessible. Because users need only their own smartphone, with no additional hardware and no body-worn sensors or physical contact during the night, the threshold to use it is low.

After each recorded night, the OSA risk estimate is presented using a familiar traffic-light scheme: green indicates an estimated AHI < 15, yellow an AHI of 15 to below 30, and red an AHI of 30 or above, corresponding to the international classification of OSA severity [18]. In line with the diagnostic guidelines for sleep-related breathing disorders in adults, an AHI ≥ 15 defines OSA requiring treatment [18]; accordingly, the application recommends a physician visit whenever the result is yellow or red.

Technically, the nocturnal sound recorded by the smartphone microphone is filtered by the application’s processing chain to the spectral ranges characteristic of respiratory activity and subjected to a noise reduction pre-processing stage. This yields a robust representation of the sound intensity generated by respiratory activity (breathing and snoring) even in acoustically unfavorable environments. From this respiratory sound-intensity signal, the algorithm derives the acoustic features used to identify respiratory activity and anomalies and to estimate the AHI. Figure 1 illustrates, for a segment containing several apneas, the correspondence between the respiratory sound intensity detected by the application and selected channels of the simultaneously recorded polysomnography (nasal flow, thoracic and abdominal effort, and oxygen saturation). Episodes of respiratory sound activity track the flow and effort signals, and the intervening pauses are followed by the expected oxygen desaturations.

Because the risk estimate is based purely on acoustic information, an appropriate acoustic environment is required: the sleeping environment must be quiet without excessive non-stationary background noise, and only a single source of breathing sounds may be present in the room. Users must therefore sleep alone, without a bed partner, and ensure a quiet setting; the application reminds them of these requirements before every measurement. Brief or intermittent extraneous sounds, such as speech, a running television, or transient noise such as passing traffic, do not impair the measurement. If the acoustic conditions do not permit a sufficient amount of breathing sound to be analyzed, the application flags the recording as “not valid” and no risk estimate is issued.

Further, the application analyzes the periodicity of the recorded sound signal within the spectral bands relevant to breathing. Recordings in which the breathing sounds are non-periodic or fall outside the range plausible for a single individual’s respiratory rate (which would be the case when a second person or a snoring pet is present) are automatically flagged as invalid and no risk estimate is issued. Such cases of non-compliance with the requirement to sleep alone are therefore excluded from the dataset (see Figure 2 for details).

The application also collects user self-reported symptoms that may indicate the presence of a sleep-related breathing disorder (Table 1). The questions follow the recommendations of international guidelines for capturing the cardinal symptoms of OSA: snoring, hypertension, witnessed apnea and excessive daytime sleepiness. Following the recommendations of the American Academy of Sleep Medicine (AASM) [18], a physician visit is recommended when at least two of questions 1–3 are answered “yes” together with excessive daytime sleepiness, defined as an Epworth Sleepiness Scale (ESS) score > 10. Users may additionally provide their age, weight and height on a voluntary basis.

### 2.2. Data Donation, Consent and Ethics

Users of the application may voluntarily donate their data for research purposes. Use of the application is independent of this decision: neither the range of functions nor the user experience depends on consent to data donation. All users who provided informed consent for the research use of their anonymized data during application onboarding between March 2023 and December 2025 were included in the present dataset. Data from users who declined consent were never transferred for research use and are not part of this dataset. The dataset consists of fully anonymized data suitable for retrospective research, with no access to personally identifiable information and no possibility of re-identification. In accordance with applicable German national regulations and institutional policies, ethics approval is not required for retrospective analyses of fully anonymized observational data, which was confirmed by the Ethics Committee of the Medical Faculty of the University of Duisburg-Essen. All data handling and analysis complied with the relevant data protection and privacy standards. The dataset was collected and provided by the manufacturer of the application (Diametos GmbH). The first author (CJ) is an employee and shareholder of Diametos GmbH and was not involved in data validation (see Author Contributions and Conflicts of Interest), which was carried out by co-authors without any financial interest in, or in-kind support from, the manufacturer.

### 2.3. Validation Against the Diagnostic Gold Standard

The diagnostic performance of Snorefox M was established in a dedicated validation study (the ESOSA study), in which the application was used in parallel with type II PSG and its severity-class detection performance was assessed against the PSG reference [19]. That study was a prospective, non-interventional, single-arm, non-randomized observational investigation conducted in accordance with the ISO standard on the clinical investigation of medical devices for human subjects (EN ISO 14155:2011 [20]); ethical approval was granted by the Ethics Committee of the Bavarian State Medical Association.

The data underlying the performance validation in Section 3.5 were not newly acquired for this work. They originate entirely from the previously published, independent ESOSA study [19], from which the present paper draws a post hoc statistical re-analysis at additional thresholds. The ESOSA study participants constitute a separate, supervised cohort and are not part of the unsupervised real-world dataset described in Section 3.1. In brief, volunteers were recruited through social media advertising and the manufacturer’s existing newsletter mailing list. To obtain a sample representative of the general population, the inclusion criteria were deliberately broad, the only restrictions being adult age, willingness to participate and the ability to use a smartphone. Individuals who self-reported a combination of symptoms suggestive of an elevated OSA risk were excluded from enrollment and advised to consult a physician for diagnostic work-up of a sleep-related breathing disorder.

Enrolled participants slept alone for one night and used Snorefox M in parallel with a PSG recorded on the same night (Sonata, Löwenstein Medical, Germany). The PSG captured the physiological signals required to score OSA severity according to the AASM criteria [21]. All PSG recordings were scored manually by two certified sleep technicians using annotation software (MiniScreen Version 5.21a, Dr. Fenyves und Gut GmbH, Rangendingen, Germany). Where the two scorers disagreed about the relevant severity threshold (AHI ≥ 15 versus AHI < 15), a third technician scored the recording and a majority vote was applied. All technicians were blinded to one another’s results and to the output of the application.

Scoring followed Version 3 of the AASM Manual for the Scoring of Sleep and Associated Events [21], with hypopneas defined using an oxygen desaturation index threshold >3%. An AHI ≥ 15 was classified as moderate OSA and an AHI ≥ 30 as severe OSA. Following Epstein et al. [22], the AHI was evaluated to the first decimal place.

### 2.4. Smartphone Coverage and Population Representativeness

Smartphone use across the general German population, broken down by age group, is summarized in Table 2 [23]. Smartphone use is near-universal among younger adults but declines with advancing age, whereas the prevalence of OSA rises with age [24]. As a result, the segment of the population in which OSA is most prevalent, older adults, is the segment least likely to be fully represented among smartphone-based screening users. The user population is therefore expected to under-represent the oldest and highest-prevalence age groups, which might tend to attenuate the observed overall prevalence relative to a population that included these groups in proportion.

For data security reasons, Snorefox M runs only on actively maintained Android and iOS versions that continue to receive security updates from their providers, ensuring that user data are handled on up-to-date, secure hardware. Together, these supported operating systems and compatible smartphone models account for approximately 75% of the smartphones in use on the German market [25].

A further bias is introduced by self-selection. Individuals with an existing interest in their sleep health, or who already suspect a sleep problem, are more likely to install and use the application than those who are unaware of nocturnal breathing problems or oblivious to the telltale symptoms of OSA.

### 2.5. Statistical Analysis

All analyses were performed in R, version 4.3.2 (R Foundation for Statistical Computing, Vienna, Austria). Sensitivity, specificity, positive predictive value (PPV) and negative predictive value (NPV) were calculated with 95% confidence intervals using standard definitions. The primary analysis population was a modified intent-to-diagnose population comprising all subjects for whom demographic baseline information, a valid Snorefox M result and a valid PSG result from the diagnostic night were available (Table 3).

The sensitivity and specificity for detecting moderate-to-severe OSA (AHI ≥ 15) were the primary endpoints of the original validation study, for which it was powered [19]. To explore the behavior of the application across further clinically relevant severity stages, we additionally analyzed its classification performance at further AHI thresholds (AHI ≥ 30, and AHI < 10). These analyses are post hoc, and the study was not powered for these thresholds. For each threshold, only subjects for whom both manual PSG scorers agreed with respect to that threshold were included; ambiguous cases and subjects lacking a valid PSG or Snorefox M result were excluded. Results are reported with 95% confidence intervals. Finally, the linear relationship between the app-derived and PSG-derived AHI was quantified with the Pearson correlation coefficient, using the mean of the two raters’ AHI values as the reference. The post hoc threshold analysis is a secondary statistical re-analysis of the independently acquired, anonymized reference scores from the ESOSA study [19]. The underlying polysomnography data were neither re-accessed nor re-scored for the present work.

In addition, the discriminative ability of the app-derived AHI was assessed by receiver operating characteristic (ROC) analysis at thresholds of AHI ≥ 10, AHI ≥ 15 and AHI ≥ 30; for each threshold, the PSG-derived AHI dichotomized at the respective cut-off served as the reference state and the continuous Snorefox M AHI estimate as the predictor, and the area under the curve (AUC) was computed as a threshold-independent summary of discrimination.

## 3. Results

### 3.1. Dataset Properties

Between March 2023 and December 2025, a total of 31,647 users who had consented to research data donation contributed a total of 239,132 measurements.

The robustness of the measurement under real-world conditions was quantified as the proportion of attempted measurements that yielded a valid analysis result. A measurement was counted as attempted when a recording was started and was not deliberately aborted within the first two hours. Recordings aborted by the user were not regarded as measurement failures and were excluded from this analysis. Across all contributed measurements, 230,730 yielded a valid analysis result, while for 8402 measurements, no result could be generated for acoustic reasons (that is, insufficient detectable breathing activity or excessive background noise), or the analysis failed because of an incomplete data upload. This corresponds to a yield of 96.5% obtained entirely without supervision, additional hardware or set-up support, and it indicates that the measurement is robust under everyday conditions. It is comparable to the rate of valid results achieved in the supervised validation study, in which the application did not yield a valid result in 3 of 150 nights (2.0%; Table 3).

Restricting the dataset to users with at least two valid nights yielded 221,820 nights of sleep screening data from 14,351 adults, corresponding to a mean of approximately 15.5 recorded nights per user (see all details in the consort chart in Figure 2).

This constitutes, for the first time, a longitudinal dataset of OSA risk screening obtained in an unsupervised, general adult population. The only inherent restriction is that users must possess a compatible smartphone and be able to operate it in their private sleeping environment. The longitudinal depth of the dataset is distributed unevenly across users. Roughly one-quarter of users (24.1%) contributed two nights, and just over half (53.8%) contributed five nights or fewer; so, the median number of recorded nights falls within the three- to five-night band. At the same time, 28.0% of users recorded more than 10 nights, 10.2% more than 30 nights, and a small group of 372 users (2.6%) more than 100 nights. The mean of approximately 15.5 nights per user is therefore driven by this long right tail; the distribution is strongly right-skewed (SD 39.8 nights, range 2–999).

This pattern is itself informative for a screening tool intended for repeated use: a substantial minority of users engage with the application over extended periods and thereby generate exactly the kind of longitudinal data that standard diagnostics cannot supply. The analysis of night-to-night variability in the AHI is the subject of dedicated work in preparation.

### 3.2. Recording Duration

Users were instructed by the app to terminate their nightly recording manually at the time of getting up. Recordings with a duration of less than two hours are considered prematurely aborted by the user and are not analyzed by the application. After eight hours of recording, the application finishes a recording automatically. Figure 3 shows the distribution of recording duration across all valid measurements. In total, 73% of all recordings are at least six hours long and therefore fulfill the minimum length criterion for a valid polysomnography recording. A minority of 6% of the recordings are shorter than four hours.

To assess whether short recording durations of <4 h systematically affect AHI results, a linear mixed-effects model with a subject-specific random intercept was used to account for repeated measurements within individuals, and within- and between-subject effects of short recording duration were separated by subject-mean centering. Only those subjects were included who contributed at least one night with a recording duration < 4 h and at least one night with a recording duration ≥ 4 h, resulting in 145,718 nights from 4916 participants.

A recording duration < 4 h was associated with a small reduction in the AHI compared with ≥4 h within the same individual (estimated difference −0.23 events per hour, 95% CI −0.37 to −0.08; *p* = 0.0029). Although the difference is statistically significant, its absolute magnitude is small, indicating only a minor systematic bias associated with recording durations below 4 h.

### 3.3. Distribution of Estimated OSA Risk in the User Base

Across all 230,730 valid measurements, the mean estimated AHI was 14.3 (median 11.8, standard deviation 11.0). The distribution across the three risk categories is shown in Table 4: 148,867 measurements (65%) were classified as green (AHI < 15), 64,684 (28%) as yellow (AHI 15–30) and 17,179 (7%) as red (AHI ≥ 30).

Taken together, 35% of all valid measurements indicated an estimated AHI ≥ 15. This proportion is higher than the prevalence of moderate-to-severe OSA reported for the general adult population, which is on the order of one in five [11]. The most plausible explanation is self-selection: as outlined above, users who choose to install and use an OSA screening application are more likely to snore, to suspect a sleep problem or to carry a higher pretest probability of OSA than the population at large. The observed distribution should therefore be read as descriptive of the screening tool user base rather than as an unbiased estimate of population prevalence.

### 3.4. User Characteristics

Beyond the nightly screening result, users could voluntarily provide certain demographic information and could complete a symptom questionnaire including the ESS (Table 1). This information is self-reported and not verified; it was available for a subgroup of 35% of the consenting users with at least one valid measurement. Descriptive statistics for these variables are therefore reported for this subgroup only and are not necessarily representative of the entire user population. The characteristics for this subgroup, together with the distribution of the estimated AHI across all measurements, are summarized in Table 5.

Users in this subgroup were concentrated in middle and older adulthood, in which OSA prevalence is highest, while still spanning the full adult age range. The mean ESS score was 7.2 (median 7) and thus below the threshold of 10 that is commonly taken to indicate excessive daytime sleepiness, which is consistent with a user population that is largely not overtly symptomatic. A detailed analysis of user demographics and the relationship between demographic factors, reported symptoms and screening results is the subject of dedicated work in preparation.

### 3.5. Performance Validation Against PSG

The performance validation against the gold standard was based on 150 subjects who underwent type II PSG in parallel with a Snorefox M measurement. All 150 subjects enrolled in the study completed the observational night, and no adverse events occurred [19]. The application failed to return a valid result in 3 cases, the PSG could not be scored because of missing SpO_2_ data in 3 cases, and insufficient EEG-defined sleep time precluded scoring in 2 cases; so, 142 subjects were included in the analysis with a valid result (Table 3).

For the primary threshold of moderate-to-severe OSA (AHI ≥ 15), Snorefox M achieved a sensitivity of 0.91 and a specificity of 0.83, with a PPV of 0.63 and an NPV of 0.97 at the observed prevalence of 24% [19]. In addition to the results published in [19], a post hoc analysis at further thresholds was performed on the original data. All results are reported in Table 6.

All PSG recordings had originally been scored by two blinded scorers, and where the two disagreed with respect to the AHI ≥ 15 threshold, a third scorer was consulted and the majority vote applied. For the post hoc analyses, the scoring was not repeated; the original PSG scores were reused to ensure consistency. As a result, for each additional threshold, subjects were excluded from the corresponding analysis whenever the two scorers disagreed with respect to that threshold; so, the number of subjects included may differ between thresholds. For the AHI ≥ 30 threshold, this applied to 5 subjects, leaving 137 subjects for analysis; for the AHI < 10 threshold, the scorers disagreed in 10 cases, leaving 132 subjects.

For severe OSA (AHI ≥ 30; *n* = 137), the application showed a sensitivity of 0.93 and a specificity of 0.97, with a PPV of 0.76 and an NPV of 0.99. For AHI < 10 (*n* = 132), the sensitivity was 0.62 with a specificity of 0.97. In other words, the application is most reliable at the clinically decisive boundaries that separate treatment-relevant from non-treatment-relevant disease (AHI 15 and AHI 30), and is less able to resolve fine gradations within the normal-to-mild range.

The true OSA prevalence in the validation sample, as determined by PSG, was 24% at the AHI ≥ 15 threshold and 9% at the AHI ≥ 30 threshold, while 59% of subjects had an AHI < 10. These proportions are somewhat higher than the corresponding estimates for the general adult population [11]. This is most plausibly explained by self-selection during recruitment: although subjects reporting a combination of overt OSA symptoms were excluded, individuals who volunteer for a sleep study are likely to carry a higher pretest probability of OSA than the population at large.

### 3.6. Correlation Between App-Derived and PSG-Derived AHI

Across the 142 subjects with a valid result from both methods, the AHI estimated by Snorefox M correlated strongly with the AHI derived from PSG (mean of the two raters): Pearson r = 0.80 (95% CI 0.74–0.86; t = 15.98, df = 140, *p* < 0.001). The coefficient of determination (r^2^ = 0.65) indicates that about two-thirds of the variance in the PSG-derived AHI is captured by the acoustic estimate. A correlation of this magnitude is conventionally regarded as strong and supports the use of the app-derived AHI for screening purposes, while the residual variance is consistent with the night-to-night and methodological variability inherent in any single-night AHI determination.

### 3.7. Receiver Operating Characteristics at Relevant Thresholds

To complement the threshold-specific operating points, receiver operating characteristic (ROC) curves were constructed for three thresholds by comparing the continuous Snorefox M AHI estimate against the PSG-derived AHI dichotomized at the respective cut-off, and the area under the curve (AUC) was used to summarize the overall discriminative performance independently of any single operating point. The resulting AUCs were 0.951 (95% CI, 0.906–0.997) for the severe-OSA threshold (AHI ≥ 30; Figure 4a), 0.925 (95% CI, 0.882–0.967) for the moderate-to-severe threshold (AHI ≥ 15; Figure 4b) and 0.877 (95% CI, 0.82–0.933) for the AHI ≥ 10 threshold (Figure 4c). By conventional criteria, an AUC above 0.9 indicates excellent discrimination and an AUC between 0.8 and 0.9 good discrimination; the app-derived AHI therefore discriminated excellently at the two thresholds that are decisive for treatment and referral (AHI ≥ 15 and AHI ≥ 30) and remained good at the lower AHI ≥ 10 threshold.

## 4. Discussion

OSA is a highly prevalent yet substantially underdiagnosed disorder with major individual and socioeconomic consequences. The dataset described here is, to our knowledge, the first and by far the largest collection of OSA risk screening data obtained from an unsupervised, self-selected general adult population. Its defining feature is longitudinal depth: with more than 220,000 nights contributed by over 14,000 users, it captures repeated measurements over time for individual users at a scale and granularity that have not previously been available.

A central question for any screening dataset is whether the underlying measurement can be trusted. The validation against PSG provides reassurance on this point: at the two clinically decisive thresholds, an AHI of 15, which separates treatment-relevant from non-treatment-relevant disease, and an AHI of 30, which defines severe disease, the application achieved high sensitivity and specificity, and the app-derived AHI correlated strongly with the PSG reference.

The screening performance should be interpreted against the reliability of the clinical reference standard itself. Manual scoring of respiratory events is subject to relevant inter-scorer variability: in a large international comparison, agreement between sleep centers on respiratory event scoring and the resulting AHI was only moderate, and disagreement was sufficient to shift individual patients across diagnostic categories [26]. Inter-scorer reliability programs have likewise documented systematic differences in respiratory event identification between scorers [27]. Home sleep apnea testing introduces additional variability: unattended studies that lack EEG-based sleep staging tend to underestimate severity because total recording time, rather than actual sleep time, is used as the denominator [28].

Any measured performance of an index test can, in principle, not exceed the accuracy of the reference standard against which it is evaluated. In the ESOSA validation, this limitation was mitigated to a certain extent by using the mean of two independent, blinded scorers as the reference. Furthermore, at the clinically relevant threshold of AHI = 15, any disagreement between the two scorers was resolved by a third blinded scorer, further improving the quality of the reference. Nonetheless, a residual ceiling remains: part of the apparent discrepancy between the app-derived AHI and the polysomnographic reference may reflect variability in the reference rather than error of the index test, and the validation metrics should be read in this light rather than as absolute measures of accuracy.

Seen against this background, i.e., considering both the agreement with the reference and the inherent imperfection of the reference itself, the sensitivity and specificity achieved by Snorefox M at the clinically decisive thresholds are consistent with the requirements of a screening context, where the primary objective is to identify individuals who should be referred for further diagnostic workup.

The principal strengths of this dataset are its large sample size, its considerable longitudinal depth, and its reliance on real-world, unsupervised recordings made in naturalistic home settings rather than in the artificial environment of a sleep laboratory. The repeated nightly sampling per user opens a window onto the night-to-night variability in OSA risk that single-night testing cannot provide, and it does so for a population that is largely invisible to conventional sleep medicine, people who are interested in their sleep health but have not yet entered the diagnostic pathway.

These strengths are counterbalanced by limitations that are intrinsic to an unsupervised, real-world dataset. The user population is self-selected and therefore biased: it over-represents individuals with an interest in or concern about their sleep and, as discussed, under-represents the oldest age groups in whom OSA prevalence is highest. The dataset is consequently not representative of the general population, and the observed severity distribution should not be read as a population prevalence estimate. Demographic and symptom information is self-reported in an unsupervised setting and is therefore expected to be noisy and incomplete. Such limitations are normal and to be expected for a dataset of this kind; they define the appropriate uses of the data, hypothesis generation, description of longitudinal patterns and triage support, rather than precise epidemiological estimation. Anthropometric measurements and detailed information on sleep architecture, body position and environmental conditions were not available, which limits any mechanistic interpretation of the observed variability. Finally, clinical outcomes and the effects of treatment were not assessed.

The reported distributions and summary measures of the estimated AHI were calculated at the measurement level, across all valid nights, rather than at the user level. Because the number of recordings per user is strongly right-skewed, with a minority of users contributing a disproportionately large number of nights, these users carry correspondingly greater weight in the aggregate figures. A user-level analysis, including the night-to-night variability in the estimated AHI and its implications for individual risk classification, is beyond the scope of this paper and should be the subject of dedicated future investigations based on this dataset. The descriptive statistics reported here should accordingly be interpreted as properties of the measurement corpus rather than as user-level estimates.

A screening tool that can be used repeatedly at low marginal cost over many nights may add information that single-night diagnostics cannot, helping to address both underdiagnosis and the night-to-night variability in the AHI. Future work linking longitudinal screening trajectories to clinical outcomes, and integrating validated digital screening into primary care and specialist referral pathways, would clarify the prognostic and health economic value of this information.

The main value of this resource lies in the analyses it makes possible, and the present paper deliberately confines itself to introducing the dataset and to demonstrating its validity compared to the diagnostic gold standard. Further investigation is being pursued by our group, including a detailed characterization of the user population and of the relationship between demographic factors, self-reported symptoms and screening results; analysis of the night-to-night variability in the estimated AHI in longitudinal, multi-night use; and an appraisal of the consequences of that variability for screening and referral pathways.

## 5. Conclusions

This work describes the first large-scale, real-world dataset of smartphone-based OSA risk screening obtained from an unsupervised, self-selected general adult population, together with a summary and post hoc extension of the validation of the underlying tool against type II PSG. Its repeated, low-threshold use has the potential to generate longitudinal information that conventional single-night testing cannot provide. Used appropriately, as a screening and triage aid rather than a diagnostic test, such a tool can complement established diagnostic pathways, help to narrow the large diagnostic gap in OSA, and support earlier referral of individuals who would otherwise remain undetected, with potential benefits for cardiovascular health at the population level. The self-selected, unsupervised nature of the data defines both its unique value and its principal constraints, and points to the integration of validated digital screening into clinical pathways and the linkage of longitudinal screening data to clinical outcomes as priorities for future research.

## Figures and Tables

**Figure 1:**
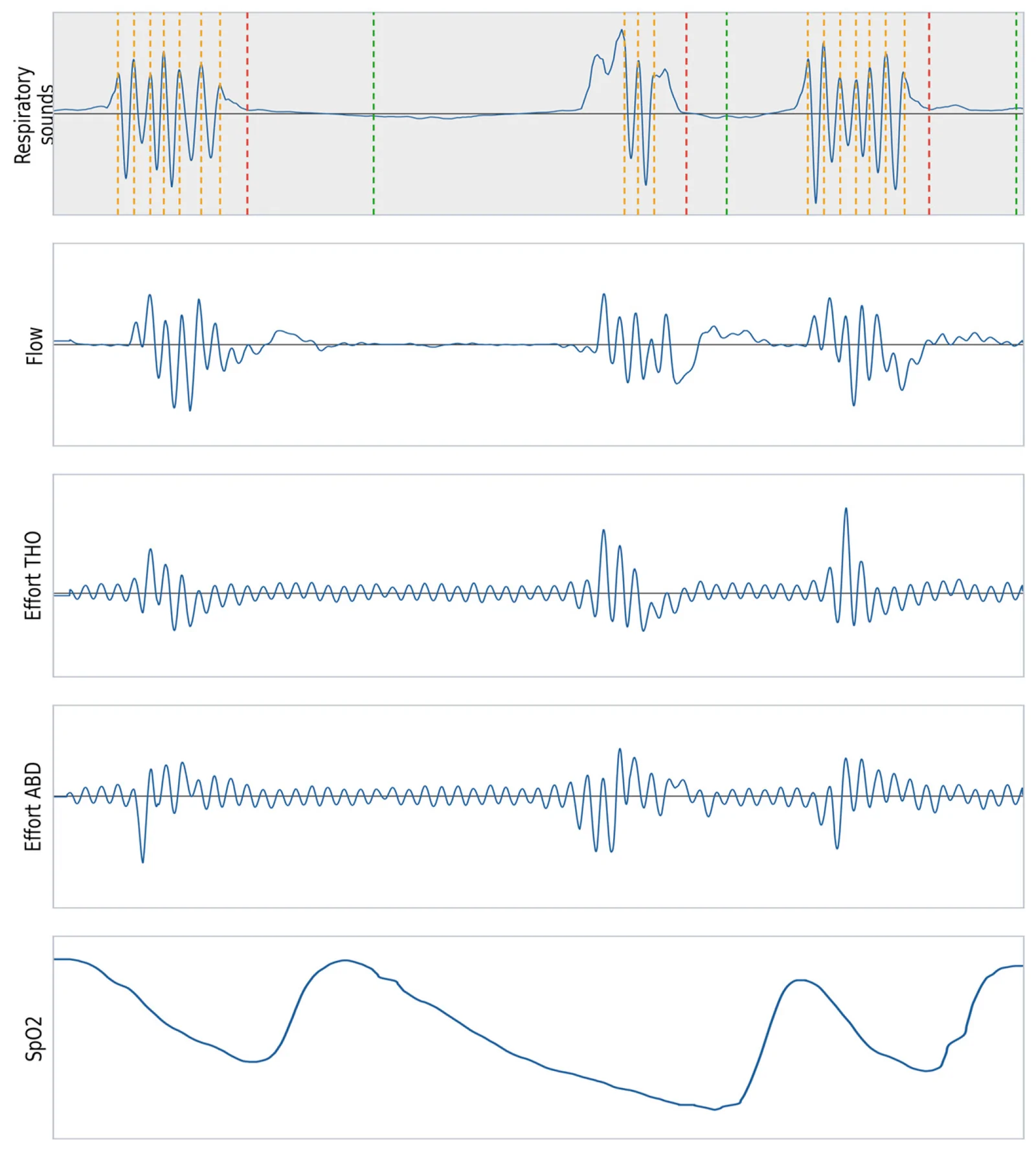
An example of the relationship between the respiratory sound detected by the application and the simultaneously recorded polysomnography channels, nasal flow, thoracic effort (Effort THO), abdominal effort (Effort ABD) and oxygen saturation (SpO_2_). The top trace shows the app-derived respiratory sound signal; colored dashed lines mark respiratory activity and events. Relative amplitudes are not to scale and have been adjusted for clarity of presentation.

**Figure 2:**
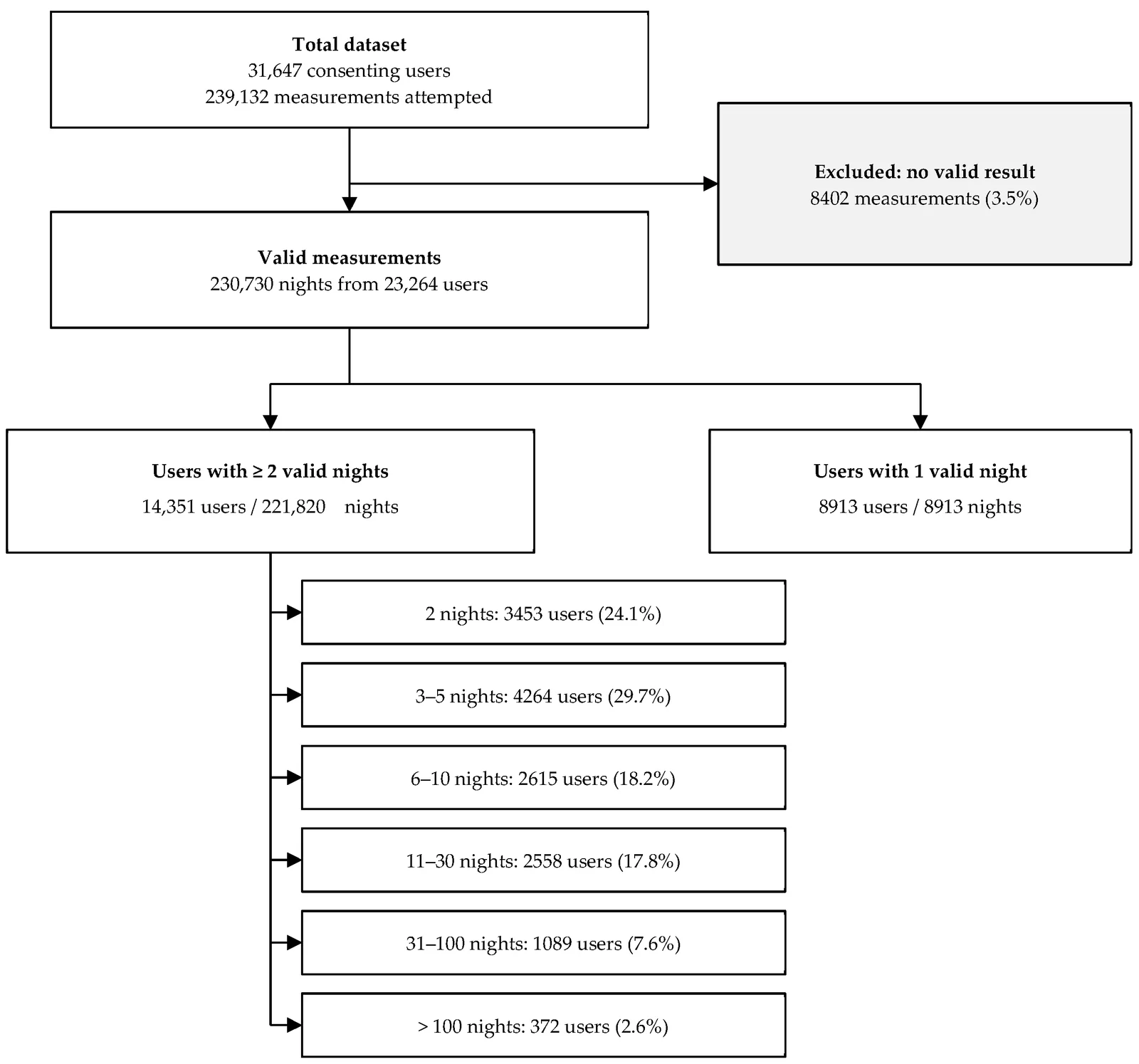
The flow of measurements and users in the screening dataset. Numbers refer to measurements between March 2023 and December 2025 by users who consented to the research use of their anonymized data. The distribution of recording nights per user is shown for the cohort contributing at least two valid nights.

**Figure 3:**
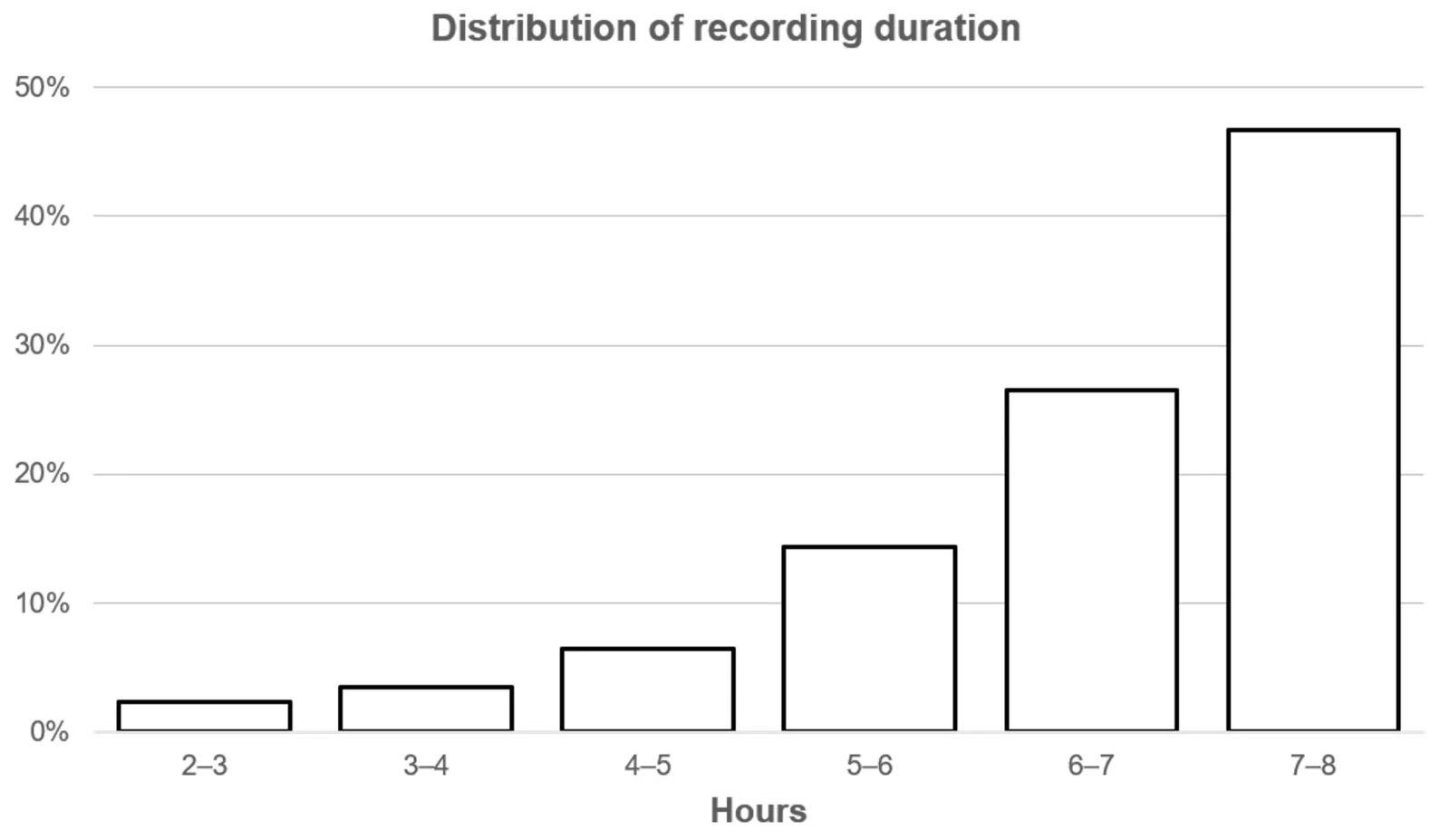
Distribution of recording duration across all valid measurements.

**Figure 4:**
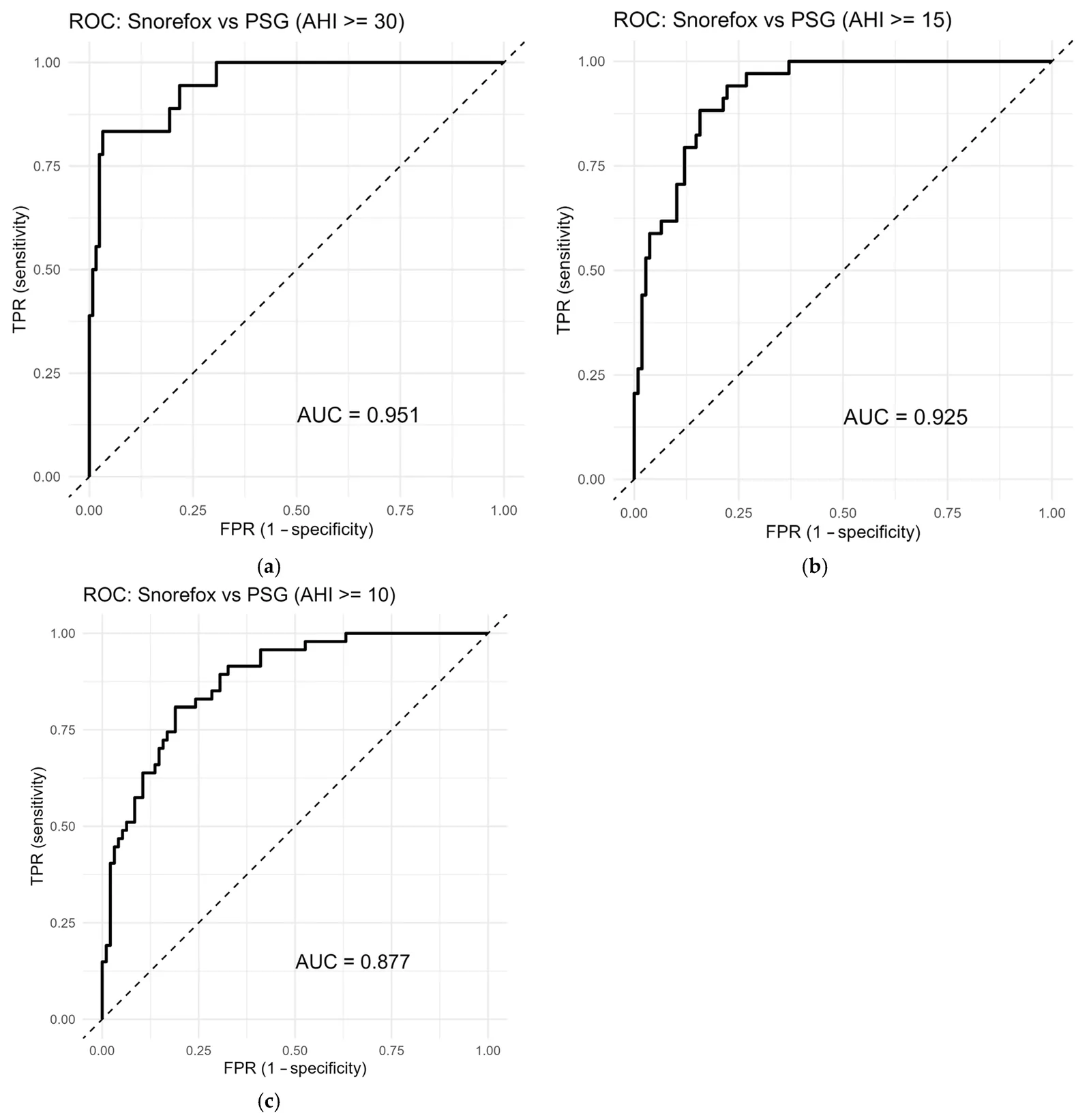
Receiver operating characteristic (ROC) curves for the detection of OSA by the Snorefox M AHI estimate against the type II PSG reference at the thresholds (**a**) AHI ≥ 30 (AUC = 0.951), (**b**) AHI ≥ 15 (AUC = 0.925) and (**c**) AHI ≥ 10 (AUC = 0.877). TPR, true positive rate; FPR, false positive rate.

**Table 1:** Pre-screening questions used by the application to self-assess OSA-related symptoms.

Category	Items
Symptom questions (yes/no)	1. Do you snore loudly and regularly (every night or almost every night)? 2. Has anyone ever noticed that you stop breathing or gasp for air in your sleep? 3. Do you have high blood pressure or are you being treated for it?
Epworth Sleepiness Scale—chance of dozing (0 = would never doze; 1 = slight; 2 = moderate; 3 = high)	Sitting and reading; watching TV; sitting inactive in a public place; being a passenger in a car for an hour; lying down in the afternoon; sitting and talking to someone; sitting quietly after lunch (no alcohol); stopping for a few minutes in traffic while driving.

A physician visit is recommended when at least two of questions 1–3 are answered “yes” and the ESS score exceeds 10.

**Table 2:** Smartphone use in the general German population by age group [23].

Age Group (Years)	Smartphone Use (%)
14–27	99
28–42	99
43–57	97
58–67	91
68–77	77
≥78	42

**Table 3:** Subjects without a study result in the validation study.

	Number of Subjects
Subjects enrolled	150
Subjects that did not complete the study	0
Snorefox M did not provide a result	3
PSG invalid result (no SpO_2_ data)	3
Insufficient sleep time according to EEG	2

**Table 4:** Distribution of measurements across OSA risk categories (all valid measurements, *n* = 230,730).

Category	AHI Range	Measurements	Share
Green	AHI < 15	148,867	65%
Yellow	AHI 15–30	64,684	28%
Red	AHI ≥ 30	17,179	7%

**Table 5:** Selected characteristics of the screening dataset. AHI statistics refer to all valid measurements; age and ESS score were available for a subgroup of users.

Variable	*n*	Mean (SD)	Min	Median	Max
Estimated AHI (events/h) ^1^	230,730	14.34 (10.99)	0.00	11.77	225.50
Age (years) ^2^	8092	54.43 (11.26)	18	55	92
ESS score ^2^	8092	7.20 (3.52)	0	7	24

AHI, apnea–hypopnea index; ESS, Epworth Sleepiness Scale; SD, standard deviation. ^1^ Across all valid measurements. ^2^ Self-reported, provided voluntarily by a subgroup.

**Table 6:** Classification performance of Snorefox M at different AHI thresholds, based on the analysis population of the ESOSA validation study (point estimate with 95% confidence interval). The AHI ≥ 15 column is the powered primary endpoint; all other columns are post hoc analyses for which the study was not powered.

Metric	AHI ≥ 15 ^1^	AHI ≥ 30	AHI < 10
*n*	142	137	132
True prevalence	0.24 (0.17–0.32)	0.09 (0.05–0.15)	0.59 (0.51–0.67)
Sensitivity	0.91 (0.76–0.98)	0.93 (0.66–1.00)	0.62 (0.51–0.72)
Specificity	0.83 (0.75–0.90)	0.97 (0.93–0.99)	0.97 (0.89–1.00)
PPV	0.63 (0.48–0.77)	0.76 (0.50–0.93)	0.96 (0.88–1.00)
NPV	0.97 (0.91–0.99)	0.99 (0.96–1.00)	0.63 (0.53–0.73)

AHI, apnea–hypopnea index; PPV, positive predictive value; NPV, negative predictive value. ^1^: Powered primary endpoint of validation study [19] (*n* = 142).

## Data Availability

The data presented in this study are available on request from the corresponding author due to commercial and data-protection considerations.
